# Kidney injury molecule-1: a novel entry factor for SARS-CoV-2

**DOI:** 10.1093/jmcb/mjab006

**Published:** 2021-01-16

**Authors:** Cheng Wan, Chun Zhang

**Affiliations:** Department of Nephrology, Union Hospital, Tongji Medical College, Huazhong University of Science and Technology, Wuhan 430022, China

Renal involvement occurs frequently among patients with coronavirus disease 2019 (COVID-19) caused by severe acute respiratory syndrome coronavirus 2 (SARS-CoV-2), and acute kidney injury (AKI) is associated with poor prognosis and mortality of COVID-19. The potential mechanisms of AKI include direct virulence of SARS-CoV-2 and indirect mechanisms such as hyperinflammation, hypercoagulation, and unspecific kidney injury factors (Gabarre et al., 2020). The existence of infective SARS-CoV-2 in kidney has been verified providing a definite evidence of renal tropism and direct invasion of SARS-CoV-2 (Braun et al., 2020). Ascertaining the mechanism of SARS-CoV-2 entry will pave the way for preventing the infection and even tackling this epidemic.

The first step for viral invasion is the binding between SARS-CoV-2 spike protein and host cell receptors. Angiotensin-converting enzyme 2 (ACE2) is the first well-confirmed receptor for SARS-CoV-2 (Shang et al., 2020). Receptor-binding domain (RBD) in spike protein directly binds to ACE2, thus promoting the virus‒cell fusion and subsequent entry. CD147 is verified as another receptor for SARS-CoV-2 mediating viral entry by endocytosis (Wang et al., 2020). The redistribution of CD147 in tubular epithelial cells from basolateral pattern to circumferential pattern likely facilitates the putative invasion of SARS-CoV-2 from the lumen (Su et al., 2021). Neuropilin-1 highly expressed in the respiratory and olfactory epithelium is also an additional host cell receptor for SARS-CoV-2, and blocking the interaction between neuropilin-1 and viral spike protein reduces SARS-CoV-2 entry (Daly et al., 2020). Besides the above, Yang et al. (2021) recently proposed kidney injury molecule-1 (KIM-1) as a new host factor.

KIM-1 is well-known as a sensitive biomarker of AKI and tubular injury in other kidney diseases. It is not detectable in normal kidney, but markedly up-regulated in proximal tubular cells upon injury. In addition, KIM-1 is a double-edged sword in the process of kidney healing and injury. On the one hand, it functions as a scavenger receptor facilitating the clearance of apoptotic and necrotic cells in tubular lumen and participates in the regeneration of injured tubules (Ichimura et al., 2008). On the other hand, chronic KIM-1 expression contributes to tubulointerstitial fibrosis and inflammation (Humphreys et al., 2013). Moreover, KIM-1 is emerging as a critical entry factor for several enveloped viruses, such as Ebola virus and Dengue virus, through direct interaction between phosphatidylserine (PtdSer) binding residues within extracellular immunoglobulin variable (IgV)-like domain and PtdSer on the viral envelope, promoting the subsequent virus internalization (Moller-Tank et al., 2014).

**Figure 1:**
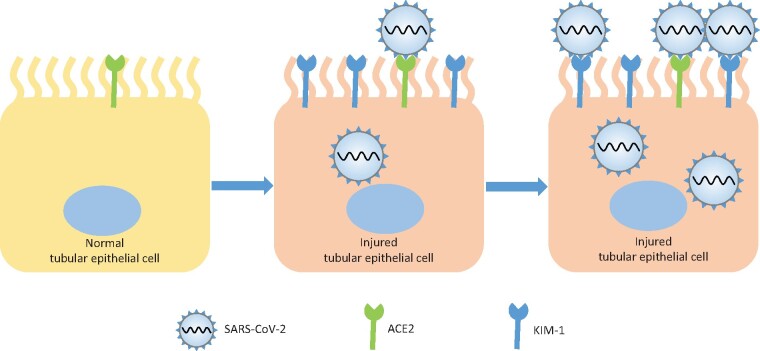
The dual role of KIM-1 in SARS-CoV-2 infection: biomarker for AKI and entry factor.

SARS-CoV-2 belongs to enveloped viruses as well. In a recent study by Yang et al. (2021), the role of KIM-1 in SARS-CoV-2 infection has been investigated. It is proposed that KIM-1 is not only a biomarker for SARS-CoV-2-associated AKI, but also a novel receptor that binds to SARS-CoV-2 RBD via IgV domain (Figure 1). Similar attachment has been revealed in SARS-CoV and Middle East respiratory syndrome coronavirus (MERS-CoV), confirming the role of KIM-1 in coronavirus invasion. It has been suggested that ACE2 and KIM-1 co-mediate the viral invasion leading to acute tubular injury, and the subsequent up-regulation of KIM-1 further promotes SARS-CoV-2 entry, thus forming a vicious cycle in the kidney. However, how KIM-1 facilitates SARS-CoV-2 internalization after binding remains to be further explored.

Another remarkable finding by Yang et al. (2021) is the designed KIM-1-derived antagonist peptide covering the two SARS-CoV-2-contacting motifs (motif 1: Leu54, Phe55, Gln58; motif 2: Trp112, Phe113) with three glycine as the flexible linker. The peptide successfully blocks the attachment between KIM-1 and SARS-CoV-2, which may help new drug discovery for SARS-CoV-2 infection (Yang et al., 2021).

In summary, Yang et al. (2021) reveals a novel virus entry route for SARS-CoV-2, KIM-1, which also acts as a biomarker for SARS-CoV-2-associated AKI. The finding of new host cell factors may provide promising targets for developing specific and effective interventions against COVID-19.
